# Long 5′ untranslated regions regulate the RNA stability of the deep-sea filamentous phage SW1

**DOI:** 10.1038/srep21908

**Published:** 2016-02-22

**Authors:** Huahua Jian, Lei Xiong, Guanpeng Xu, Xiang Xiao, Fengping Wang

**Affiliations:** 1 State Key Laboratory of Microbial Metabolism, School of Life Sciences and Biotechnology, Shanghai Jiao Tong University, Shanghai, PR China; 2 State Key Laboratory of Ocean Engineering, School of Naval Architecture, Ocean and Civil Engineering, Shanghai Jiao Tong University, Shanghai, PR China

## Abstract

Virus production in the deep-sea environment has been found to be high, and viruses have been suggested to play significant roles in the overall functioning of this ecosystem. Nevertheless, little is known about these viruses, including the mechanisms that control their production, which makes them one of the least understood biological entities on Earth. Previously, we isolated the filamentous phage SW1, whose virus production and gene transcription were found to be active at low temperatures, from a deep-sea bacterium, *Shewanella piezotolerans* WP3. In this study, the operon structure of phage SW1 is presented, which shows two operons with exceptionally long 5′ and 3′ untranslated regions (UTRs). In addition, the 5′UTR was confirmed to significantly influence the RNA stability of the SW1 transcripts. Our study revealed novel regulation of the operon and led us to propose a unique regulatory mechanism for Inoviruses. This type of RNA-based regulation may represent a mechanism for significant viral production in the cold deep biosphere.

Viruses, particularly bacteriophages, which are believed to be the most abundant biological agents in the ocean^1^, play an important role in global biogeochemical cycles, deep-sea metabolism and the overall functioning of the marine ecosystem^2^. Although lysogeny is thought to be the primary viral proliferation mode in the deep biosphere^3^, a detailed characterization of the viral genes that are expressed in this ecosystem is lacking. Because environmental conditions such as high pressure and low temperature are important for most aspects of the deep-sea ecosystem, it is expected that special regulatory elements for regulating phage gene expression are also present.

Filamentous phages (Inovirus), which contain a circular ssDNA genome, belong to the Inoviridae family of the Inovirus and Plectrovirus genera. Filamentous phages predominantly infect a variety of gram-negative bacteria, and in contrast to most bacteriophages, their secretions do not induce bacterial lysis. As temperate phages, filamentous phages affect the host’s physiological characteristics, such as growth, fragility, acid resistance, swarming motility, toxin production and biofilm formation^4^,^5^,^6^,^7^,^8^,^9^. Moreover, these phages have been shown to be key elements in lateral gene transfer and to be a primary evolutionary force in some pathogenic bacteria^10^,^11^,^12^,^13^. Recently, filamentous phages were identified in subsurface sediment, which suggests that they are linked to the carbon biogeochemical cycle and community structure^14^. In addition, the *Pseudoalteromonas* phage f327 was demonstrated to be prevalent in Arctic sea ice and to confer an advantage for host survival in the cold ecosystem^15^. Currently, the understanding of this type of virus is based on the well-studied *Escherichia coli*-infecting Ff phages (f1, M13, fd) and the *Vibrio cholera*-infecting CTXΦ phage^16^. Previously, we isolated the filamentous phage SW1 from a deep-sea bacterium, *Shewanella piezotolerans* WP3 (hereafter referred to as WP3), which was isolated from west Pacific deep-sea sediment at a water depth of 1914 m^17^,^18^. SW1 was shown to have a significant influence on the swarming ability of WP3; thus, SW1 may play an important role in adjusting the fitness of the bacterial host in the deep-sea environment^9^. In a previous study, we observed that SW1 was actively produced at low temperature. When the growth rate of WP3 decreased when cultivated at 4 °C rather than at its optimal growth temperature of 20 °C, the production of the phage SW1 was significantly induced at 4 °C compared with at 20 °C^17^. This cold induction was found to occur at the transcriptional level^19^. However, the mechanism that regulates SW1 production remains unknown.

In this study, we characterized the operon structure of SW1 and analyzed the intergenic region of key phage genes. The SW1 genome was demonstrated to include two operons, and exceptionally long 5′ and 3′UTRs were indentified. Moreover, the phage-borne regulator was shown to be able to bind to the promoter region of *fpsA* which encoded a replication protein of SW1. Interestingly, the RNA decay assay indicated that the two long 5′UTRs regulated the RNA stability of phage SW1. Taken together, our data showed that SW1 possesses a novel regulatory system, which is significantly different from the *Vibrio* phage CTXΦ, and may provide a mechanism for significant viral production in the deep-sea environment.

## Results

### The SW1 genome includes two operons

**Figure 1:**
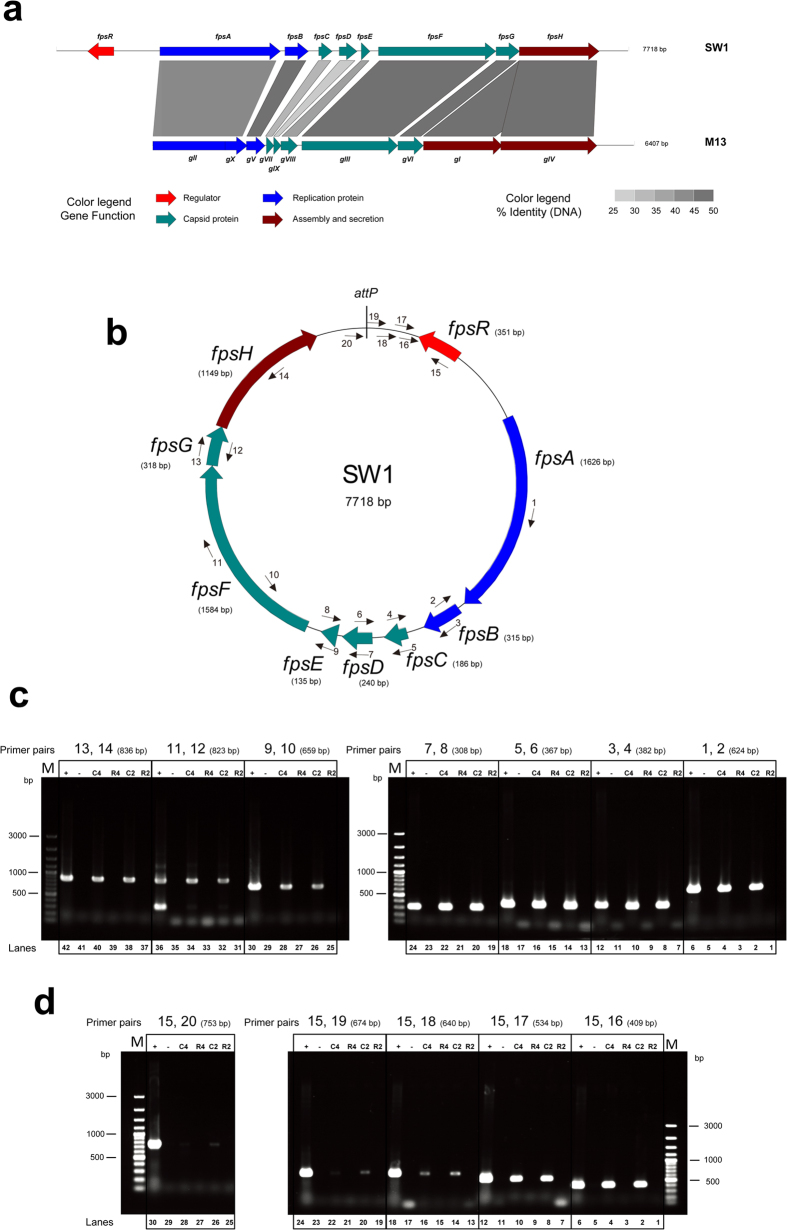
Characterization of the operon structure of SW1. (**a**) Comparative genome analysis of SW1 and M13. The scale at the top of the genome is in base pairs. Each arrow represents an ORF, with the colour representing the function of the encoded protein that is indicated in the legend. Percent identities (nucleic acids) between adjacent genomes are coloured as outlined on the bottom of the figure. (**b**) The genome map of SW1 and the primer pairs spanning across adjacent SW1 genes were used to determine whether the two genes were co-transcribed. (**c**) Identification of the co-transcription of SW1 genes using RT-PCR. (**d**) Identification of the 3′UTR downstream of *fpsR*. The 3′UTR downstream of *fpsR* was confirmed by PCR using 5 primer sets at different positions. The different templates used for each co-transcription confirmation are presented as: +, WP3 genomic DNA (positive control); -, ddwater (negative control); C4, cDNA of 4 °C; R4, RNA of 4 °C; C2, cDNA of 20 °C; R2, RNA of 20 °C. The primer pairs used in each assay are indicated as numbers: fpsA-B For/Rev (1, 2); fpsB-C For/Rev (3, 4); fpsC-D For/Rev (5, 6); fpsD-E For/Rev (7, 8); fpsE-F For/Rev (9, 10); fpsF-G For/Rev (11, 12); fpsG-H For/Rev (13, 14); fpsRD1 For/Rev (15, 16); fpsRD2 For/Rev (15, 17); fpsRD3 For/Rev (15, 18); fpsRD4 For/Rev (15, 19); fpsRD5 For/Rev (15, 20). The resulting amplicons were analyzed by electrophoresis through 1.0% agarose gels with Gel-Red staining.

SW1 contains 9 ORFs, 8 of which are oriented in one direction (*fpsA, fpsB, fpsC, fpsD, fpsE, fpsF, fpsG, fpsH*), and the last ORF, *fpsR*, encodes a putative repressor in the reverse direction^17^,^18^. A comparative genome analysis indicated that SW1 shares a similar gene order and high DNA identity with M13, except for the absence of one ORF encoding a phage assembly protein in the SW1 genome (Fig. 1a). We designed a series of primer pairs to identify whether these genes were co-transcribed (Fig. 1b and Supplementary Table S1). WP3 RNA samples from the 20 °C and 4 °C cultures were reverse-transcribed, and PCR was performed. All 8 genes oriented in the same direction were found to be co-transcribed at both temperatures and, thus, to reside in a single transcriptional operon directed by the *fpsA* promoter (*P*_*A*_) (Fig. 1c). Five primer pairs targeting sites located downstream of the coding region of *fpsR* were used to verify the transcription stop site. An amplified band was unexpectedly observed (lane 20 and 22 in Fig. 1d), though the primer was 399 bp from the termination codon of *fpsR*. This result suggested the presence of a long 3′ untranslated region (UTR) with a length of more than 399 bp (Fig. 1d).

### SW1 genes have exceptionally long 5′ UTRs

**Figure 2:**
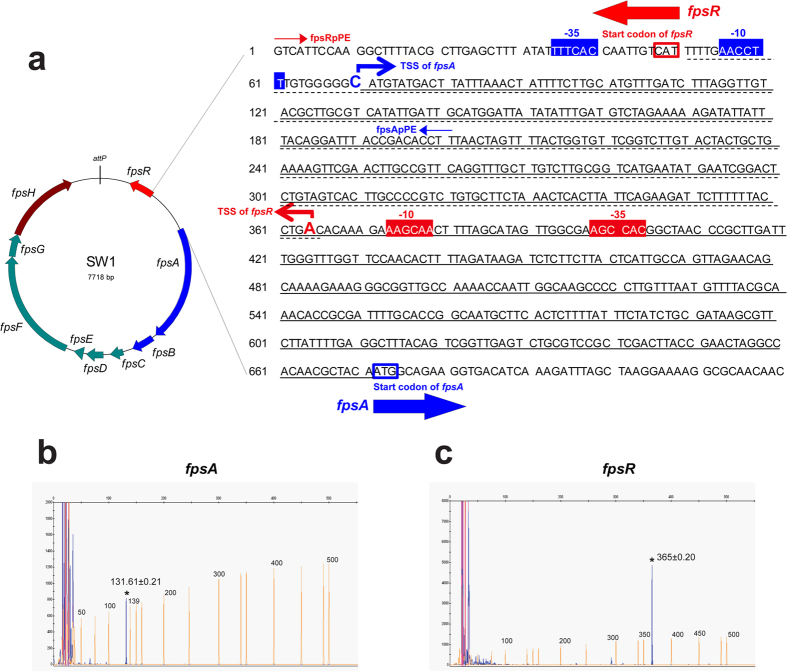
Experimental identification of the SW1 regulatory region. (**a**) Sequence of the *fpsA*-*fpsR* intergenic region of SW1. The transcription start sites of *fpsA* and *fpsR* genes are marked with large letters and angled arrows. Shaded sequences with blue and red colours are −35/−10 consensus elements of *fpsA* and *fpsR*, respectively. The start codons are boxed, and the long 5′UTRs of *fpsA* and *fpsR* are underlined with solid and dashed lines, respectively. The oligonucleotides used in the primer extension reactions are fpsApPE and fpsRpPE, which are indicated as blue and red arrows, respectively. (**b**,**c**) Determination of the transcriptional start sites of *fpsA* and *fpsR*, respectively. The red peaks are the GeneScanR-500 ROX^TM^ internal lane standards, and the size of each peak is shown (in base pairs). The blue peak labelled with asterisks in each panel is the primer extension product. The experiment was performed four times, with similar results obtained each time; the standard deviations are demonstrated.

The transcription start sites of *fpsA* and *fpsR* were characterized via primer extension of Carboxyfluorescein (FAM)-labeled cDNAs. The distance between the transcription initiation and translation start sites for *fpsA* and *fpsR* were determined to be 601 bp and 314 bp, respectively (Fig. 2b,c). Clearly, long 5′UTRs were present upstream of both *fpsABCDEFGH* and *fpsR*. Interestingly, double-stranded RNA (with a length of 295 bp) likely formed due to base pairing in the overlapping region between the UTRs (Fig. 2a). It is likely that SW1 gene transcription and production are regulated by these 5′UTRs, as the 160-nt 5′UTR of *cspA* has been identified as a major factor in the cold-induced genetic switch of the cold-shock response in *E. coli*^20^,^21^,^22^.

### FpsR binds to the promoter of *fpsA* while not to *fpsR* promoter

**Figure 3:**
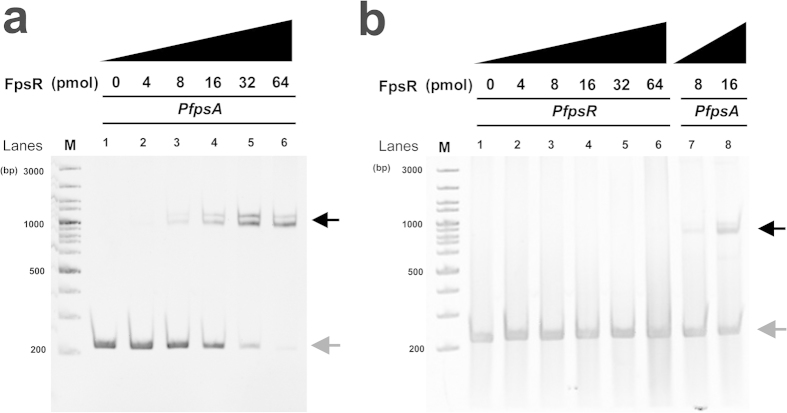
Binding of FpsR to the promoter of *fpsA* (**a**) and *fpsR* (**b**). The DNA probe was pre-incubated with increasing concentrations of purified FpsR protein as indicated. The black and grey arrows indicate the shifted DNA-protein complexes and free DNA, respectively.

The phage SW1-borne *fpsR* gene encodes a 13-kDa protein containing an N-terminal helix-turn-helix (HTH) DNA-binding motif similar to the HTH motif present in the Cro/cI superfamily of repressors; however, in contrast to the λ phage repressor CI^23^, FpsR lacks a C-terminal protease domain and cannot undergo protein autoproteolysis^17^. To elucidate its regulatory function, the *fpsR* gene was cloned into the expression vector pET-24b, and the binding of FpsR to the *fpsA* promoter (*PfpsA*) and *fpsR* promoter (*PfpsR*) was assessed using an electrophoretic mobility shift assay (EMSA) (Fig. 3). Two shifted bands were observed in a 6% nondenaturing polyacrylamide gel after FpsR and *PfpsA* were mixed at a molar ratio of 8:1 (lane 3 in Fig. 3a); when the molar ratio was increased to 64:1, the bands were completely shifted (lane 6 in Fig. 3a). However, the EMSA demonstrated that FpsR cannot binds to *PfpsR*, even at a high concentration (Fig. 3b). These results indicated that FpsR may regulate SW1 gene transcription by directly binding to the promoter region of *fpsA*.

### Proposed regulatory model for SW1

**Figure 4:**
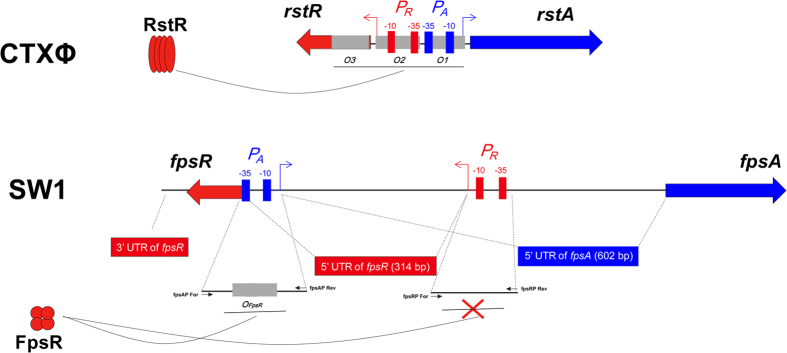
Comparison of the SW1 promoter and regulatory structure with the filamentous phage CTXФ. For CTXФ, the transcription directions of the replication gene *rstA* and the repressor gene *rstR* are reversed and share a compact regulatory region, which harbours 3 RstR binding sites. For SW1, the transcription directions of *fpsA* and *fpsR* are arranged in an uncommon opposite direction. The regulatory region contains two long 5′UTRs with overlapping portions, and a long 3′UTR is found downstream of the *fpsR* coding region. In addition, the phage-encoded regulator FpsR is able to bind to the promoter region of *fpsA* gene while not to *fpsR* gene. The primer pairs used in EMSA experiment are indicated by black arrows.

Based on these results, the structure of the regulatory region of SW1 was shown to be largely different from that of the well-documented filamentous phage CTXФ (Fig. 4). In contrast to the 138-bp ig-2 in CTXФ, the intergenic region of SW1 is 621 bp in length and contains two long 5′ UTRs that share a 295-bp overlapping 5′UTR, which is absent in CTXФ^24^. Interestingly, the *fpsA* promoter partially occupies the coding region of *fpsR*, thus suggesting a novel mechanism of transcriptional regulation, which is responsible for the interaction between RNA polymerases of transcription from *PfpsA* and *PfpsR*. Moreover, although the phage SW1-borne regulator FpsR was shown to bind to *fpsA* promoter, it cannot binds to *fpsR* promoter, indicating the absence of self-feedback inhibition of *fpsR* expression in SW1.

### The long 5′UTRs regulate the RNA stability of *fpsA*

**Figure 5:**
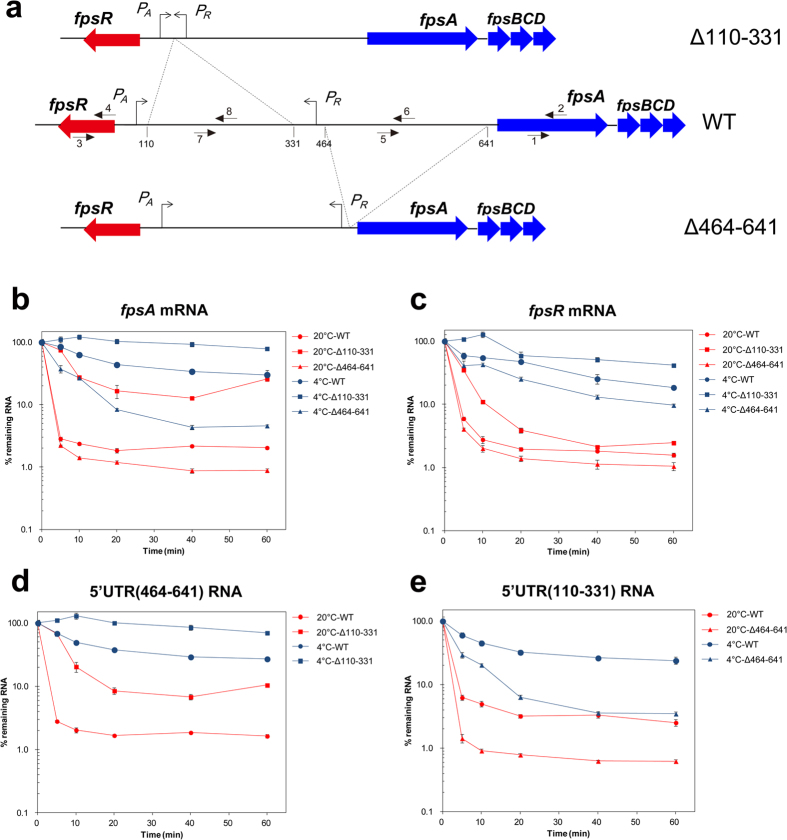
The long 5′UTR and RNA stability of SW1. (**a**) Schematic representation of the regulatory region of pSW2 (WT) and 5′UTR deletion vectors (pSW2Δ110–331 and pSW2Δ464-641). The transcription start sites are indicated with arrows, and the deletion regions are shown with dotted lines. The black arrows with numbers represent the primer pairs used for qPCR: fpsARTFor/Rev (1, 2); fpsRRTFor/Rev (3, 4); fpsA5URTFor/Rev (5, 6); fpsAR5URTFor/Rev (7, 8). (**b**,**c**) mRNA decay assay of *fpsA* and *fpsR* at 20 °C and 4 °C, respectively. (**d**,**e**) RNA decay assay of 5′UTR of 464–641 and 110–331 at 20 °C and 4 °C, respectively. The quantity of RNA at different time points was determined by reverse transcription qPCR. The data shown represent at least two independent experiments, and the error bars indicate the standard deviations of 4 replicates.

To validate the involvement of post-transcriptional regulation in the cold induction of SW1, an RNA stability assay of the *fpsA* and *fpsR* genes was performed using the *E. coli*-*Shewanella* shuttle vector pSW2, which was constructed based on the replicative form of SW1 and contained the complete sequence of *fpsR-fpsD*^25^. Intriguingly, the results revealed that the mRNA of both genes was significantly more stable at 4 °C than at 20 °C, indicating that post-transcriptional regulation involving RNA decay was an important factor in the cold induction of SW1 (Fig. 5b,c). Two derivatives of pSW2, pSW2Δ110-331 and pSW2Δ464-641, in which the overlapping region and the *fpsA* specific region of the long 5′UTR was removed, respectively (Fig. 5a), were constructed. The RNA decay assay demonstrated that the deletion of these UTRs exerted a stronger influence on the RNA stability of *fpsA* than on that of *fpsR* (Fig. 5a,c). Interestingly, the overlapping 5′UTR negatively affected the stability of *fpsA* mRNA, whereas the deletion of the *fpsA*-specific region decreased the RNA stability of *fpsA* (Fig. 5b), suggesting opposing roles of different regions of this long 5′UTR. To confirm the relationship between these two UTR fragments, the RNA stability was assessed. The results indicated that these UTRs also exhibited temperature-dependent characteristics, displaying a higher stability at the lower temperature (4 °C). As expected, the deletion of one fragment significantly affected the RNA stability of the other (Fig. 5d,e). Taken together, our data showed that the mRNA decay rates for *fpsA* and *fpsR* were significantly different at different temperatures and that the 5′UTR significantly influenced this process, suggesting the participation of long 5′UTRs in the thermoregulation of SW1 production.

## Discussion

Previously, the regulatory mechanism of filamentous phage CTXΦ has been extensively investigated^26^. The promoter of *rstA* (encoding replication protein) is adjacent to the promoter of *rstR* (encoding repressor) in CTXΦ, and the transcription process will not interact with each other after transcription initiation^27^. However, the two promoters (*PfpsA* and *PfpsR*) are separated from each other in SW1 (Fig. 4), then RNA polymerases for *fpsA* and *fpsR* transcription will encounter during the transcription because of their locations and opposite direction. In addition, the phage-borne repressor RstR in CTXΦ has been shown can binds to of the promoter of *rstR*, which formed a self-feedback inhibition of expression of RstR^26^,^28^. However, no binding was observed between SW1 encoded regulator FpsR and *PfpsR* (Fig. 3b), suggesting there is no self-feedback inhibition of FpsR. Otherwise, a novel mechanism responsible for the self-regulation of *fpsR* expression is exist, and it is significantly different from the case of RstR.

Virus production in benthic deep-sea environments has been found to be high, and viral decomposition was demonstrated to provide an important contribution for the functioning this ecosystem, which plays important roles in global geochemical cycles^2^,^29^. Meanwhile, the virus-to-cell ratios are high, which indicates an ongoing viral production in the marine deep biosphere^30^. It has recently been argued that as the cells in the deep biosphere are nearly non-proliferative^31^, viral attack may be insignificant for cell death. Temperate phages, which do not kill the microbial cells, may be more abundant (such as in the deep-sea hydrothermal plumes^32^) and play more important roles in the evolution of the community^33^,^34^. Although some phages have been isolated and identified, our current understanding of bacteriophages in the deep biosphere is still rather limited, particularly the genetic switch and induction factors. In this study, we characterized a novel promoter structure and proposed an RNA-based regulatory model for deep-sea filamentous phage SW1 production, which is significantly different from other well-known temperate phages^26^.

Regulatory RNAs, including 5′ and 3′UTRs, adjacent to the coding sequence are regulatory effectors that can influence protein expression and function in response to external cues, such as temperature, pH and metabolite levels. The 116-nt 5′UTR of *prfA* in *Listeria monocytogenes* and the 160-nt 5′UTR of *cspA* in *Escherichia coli* are the most well-known RNA thermosensors in prokaryotes^20^,^35^. A long 5′UTR has been found to contribute to mRNA stability in bacteria and Archaea^36^,^37^, and recent advances in *Listeria* and *Bacillus* transcriptome analyses have revealed regulatory roles for the 3′UTR in 3′ end-directed RNA degradation and lysine riboswitch function, respectively^38^,^39^. RNA has been considered to be more suitable for regulating gene expression than proteins due to its rapid synthesis and low energy cost^40^. This low energy cost should be especially crucial for microorganisms living in the deep biosphere, which has been characterized as an extremely low-energy environment^41^. The report of long 5′ and 3′UTRs in a bacteriophage is rather limited. Further investigation of the RNA-based mechanisms that regulate filamentous phages will broaden our understanding of the induction and survival strategies used by viruses in the habitat of the low-temperature deep biosphere.

## Methods

### Strains and growth conditions

All bacterial strains and plasmids used in this study are listed in Table 1. *Shewanella piezotolerans* WP3 was isolated from deep-sea sediment samples in our laboratory^42^,^43^. In this study, WP3 was normally cultured in modified marine 2216E medium (5 g/l tryptone, 1 g/l yeast extract, 0.1 g/l FePO_4_, 34 g/l NaCl) aerobically, with shaking at 200 rpm at different temperatures as indicated in the text. *E. coli* strains WM3064, BL21 was incubated in Luria-Bertani (LB, 10 g/l tryptone, 5 g/l yeast extract, 10 g/l NaCl) media at 37 °C. The antibiotic chloramphenicol (Cm) (Sigma, St Louis, USA) was added to the medium at 25 μg/ml and 12.5 μg/ml for *E. coli* and *Shewanella* strains, respectively, when required. Ampicillin (Sangon, Shanghai, China) was used at 100 μg/ml. The growth of the WP3 strains was determined using turbidity measurements at 600 nm with 2216E.

### Construction of vectors with UTR deletion

Construction of vectors harbouring the SW1 5′ UTR deletion fragment was performed using the *E. coli-Shewanella* shuttle vector pSW2 which was constructed based on the replicative form of SW1 and contained the complete sequence of *fpsR-fpsD*^25^. Briefly, two primer pairs located in the UTR region of SW1 were used to amplify the whole sequence of pSW2, except for the overlapping (110–331) and *fpsA* (464–641) UTR fragments, respectively. The PCR products were digested with *Apa*I and then self-ligated, yielding pSW2Δ110–331 and pSW2Δ464–641. The pSW2 and derived vectors were transformed into WM3064, which is a DAP (DL-α,ε-diaminopimelic acid) auxotroph strain. The transformants were confirmed by enzyme digestion and DNA sequencing. The vectors were introduced into WP3 by two-parent conjugation. The transconjugant was selected by chloramphenicol resistance and verified by PCR and enzyme digestion.

### RNA extraction and real-time qPCR

Total RNA was isolated with TRI reagent-RNA/DNA/protein isolation kit (MRC, Cincinnati, USA) according to the manufacturer’s instructions. The RNA samples were treated with DNase I at 37 °C for 1 h and then purified with RNeasy Mini Kit. The quantity and quality of RNA was evaluated with a UV spectrophotometer (Thermo Fisher, Waltham, USA) and agarose gel electrophoresis prior to the experiments. The purified RNA samples were used to synthesise cDNA with the RevertAid First Strand cDNA Synthesis Kit (Fermentas, Maryland, USA) following the manufacturer’s instructions. The primer pairs for the selected genes for real-time qPCR (qPCR) were designed using Primer Express software (ABI). PCR cycling was conducted using 7500 System SDS software (ABI, Foster City, USA) in reaction mixtures with total volumes of 20 μl containing 1 × SYBR Green I Universal PCR Master Mix (ABI, Foster City, USA), 0.5 μM each primer, 1 μl cDNA template. In this method, the amount of target was normalised to that of the reference gene (16S rRNA) relative to the calibrator (The amount of RNA at 0 min, was set as 100%). The 16S rRNA of WP3 was shown to be stable under the test conditions (Supplementary Fig. S1). qPCR assays were performed in triplicate for each sample, and a mean value and standard deviation were calculated for the relative RNA expression levels.

### Protein expression and purification

The expression plasmids were constructed using the expression vector pET-24b (Novagen, Madison, WI, USA). The coding region of the *fpsR* gene was PCR amplified from WP3 genomic DNA with *pfu* DNA polymerase using the primer pair fpsRHisFor/Rev. The PCR product was gel purified and then ligated into the pET-24b vector at the *Bam*HI and *Hind*III sites. *E. coli* C41(DE3) cells were transformed with this recombinant plasmid and selected on LB medium containing kanamycin. The positive clones were confirmed by enzyme digestion and DNA sequencing. C41 cells harboring pET-24b-*fpsR* were propagated in 5 ml of LB with kanamycin overnight at 37 °C. The bacteria were then inoculated in 1000 ml of fresh LB supplemented with kanamycin (50 μg/ml) and rotated at 200 rpm at 37 °C. IPTG (0.5 mM) was added when the culture was in exponential phase (OD_600_ = 0.8~1.0). The bacteria were sedimented by centrifugation at 7700 × g for 10 min at 4 °C and suspended in 10 ml of binding buffer (150 mM NaCl, 20 mM imidazole, 20 mM Tris-HCl, pH 8.0). The suspension was then sonicated on ice with a microtip probe for 10 min; during this period, each 10 s sonication was separated by an interval of 20 s. The bacterial lysates were centrifuged at 10000 × g for 20 min at 4 °C, and His-tagged proteins were purified from the soluble fraction with Ni Sepharose High-Performance resin by gravity flow according to the manufacturer’s instructions (GE Healthcare, Milwaukee, USA). The protein was eluted in elution buffer (150 mM NaCl, 500 mM imidazole, 20 mM Tris-HCl, pH 8.0), and imidazole was removed using HiTrap desalting columns (GE Healthcare, Milwaukee, USA). The purity of the protein was examined by SDS-PAGE, and protein concentrations were determined by the Bradford assay with bovine serum albumin (BSA) as the standard.

### Electrophoretic mobility shift assay (EMSA)

DNA probes were generated by PCR using primers listed in Supplementary Table S1 and purified with a cycle pure kit (Omega Bio-Tek, Norcross, USA). These fragments were mixed with different concentration of purified protein for 30 min at 20 °C. The 20 μl reaction mixture contained 40 mM KCl, 12.5 mM Tris (pH 7.5), 125 μM MnCl_2_, 1.25 mM MgCl_2_, 5% glycerol (v/v), 0.5 mM DTT, 5 μg/ml BSA and 5 ng/μl poly dIdC. Protein-DNA complexes were resolved in a 6% nondenaturing polyacrylamide gels at 20 °C with 0.5 × TBE (Tris-borate-EDTA buffer) as the running buffer. The DNA was stained with GelRed (Biotium, USA) and visualised by a gel imaging system (Tanon, Shanghai, China).

### Primer extension

The primer extension assay was carried out with the method described by Lloyd^44^, with some modifications. Briefly, 5′ FAM-labelled primers fpsApPE and fpsRpPE (Supplementary Table S1, final concentration at 10 nM) was added to 20 μg of purified total RNA, the final volume was adjusted to 20 μl using DEPC treated water and the samples were heated at 70 °C for 5 min before being chilled on ice for 20 min. The tubes were subsequently incubated at 58 °C for 20 min and then cooled at room temperature for 15 min. First-strand cDNA synthesis was performed using the AMV RT enzyme (Fermentas, Maryland, USA) according to the manufacturer’s instructions. After an initial reverse-transcription step, the 30 μl sample was used as the template for the second round RT-PCR following the addition of the reaction component. The mixture were treated with RNase A (Fermentas, Maryland, USA) at 37 °C for 30 min before being purified with Microcon YM-10 (Millipore, Billerica, USA). FAM-labelled cDNAs were dissolved in formamide and mixed with 0.5 μl of the Genescan-500 Rox internal lane standard (ABI, Foster City, USA). After the sample was heated at 95 °C for 4 min, electrophoresis was performed using an ABI 3130 XL Genetic Analyzer, and the DNA fragments were sized using the GeneMapper software, version 3.0 (ABI, Foster City, USA).

### mRNA decay assay

WP3 cells were grown in 2216E medium to the mid-exponential phase (OD_600_ = 1.2) at 20 °C and 4 °C. Rifampicin was added to the cultures to a final concentration of 1 mg/ml immediately to terminate mRNA synthesis. Two millilitres of the cultures were withdrawn at different time points (2.5 min, 5 min, 10 min, 20 min, 40 min, 60 min) after the addition of rifampicin, harvested by rapid centrifugation (15,000 × g, 10 s, 4 °C) and stored in liquid nitrogen until RNA was extracted. The transcript abundance of the particular genes of SW1 at the different temperatures was determined by qPCR. Excel software (Microsoft, USA) was used to perform data analysis.

## Additional Information

**How to cite this article**: Jian, H. *et al.* Long 5' untranslated regions regulate the RNA stability of the deep-sea filamentous phage SW1. *Sci. Rep.* **6**, 21908; doi: 10.1038/srep21908 (2016).

## Supplementary Material

Supplementary Information

## Figures and Tables

**Table 1:** Bacterial strains and plasmids used in this study.

Strain or plasmid	Description	Reference or sources
*E. coli* strain		
WM3064	Donor strain for conjugation; Δ*dapA*	45
C41(DE3)	Recombinant protein expression host	GE healthcare
*S. piezotolerans* WP3 strains		
WP3	Wild-type strain; GenBank accession no. CP000472	Laboratory stock
WP3Δ4RE	Wild-type WP3 strain with 4 restriction enzymes gene deletions	Laboratory stock (unpublished work)
WP3Δ4RE-WT	WP3Δ4 strain harboring pSW2	This work
WP3Δ110–331	WP3Δ4 strain harboring pSW2Δ110-331	This work
WP3Δ464–641	WP3Δ4 strain harboring pSW2Δ464-641	This work
Plasmids		
pRE112	Allelic-exchange vector	46
pET-24b	His-tag protein expression vector	Novagen
pET-24b-*fpsR*	pET-24b containing the coding region of *fpsR* gene	This work
pSW2	Chl R, derivative from filamentous bacteriophage SW1	Laboratory stock
pSW2Δ110–331	pSW2 with deletion of the fragment between 110 and 331 in 5′UTR of *fpsA* gene	This work
pSW2Δ464–641	pSW2 with deletion of the fragment between 464 and 641 in 5′UTR of *fpsA* gene	This work
