# Supplementary material for: Long 5′ untranslated regions regulate the RNA stability of the deep-sea filamentous phage SW1
Source: Sci Rep. 2016 Feb 22;6:21908. doi: 10.1038/srep21908 (PMC4762005; doi:10.1038/srep21908)
Supplement: Supplementary Information [file srep21908-s1.pdf]

Supplementary Information

Long 5' untranslated regions regulate the RNA stability of the deep-sea filamentous phage SW1

Huahua Jian, Lei Xiong, Guanpeng Xu, Xiang Xiao, Fengping Wang

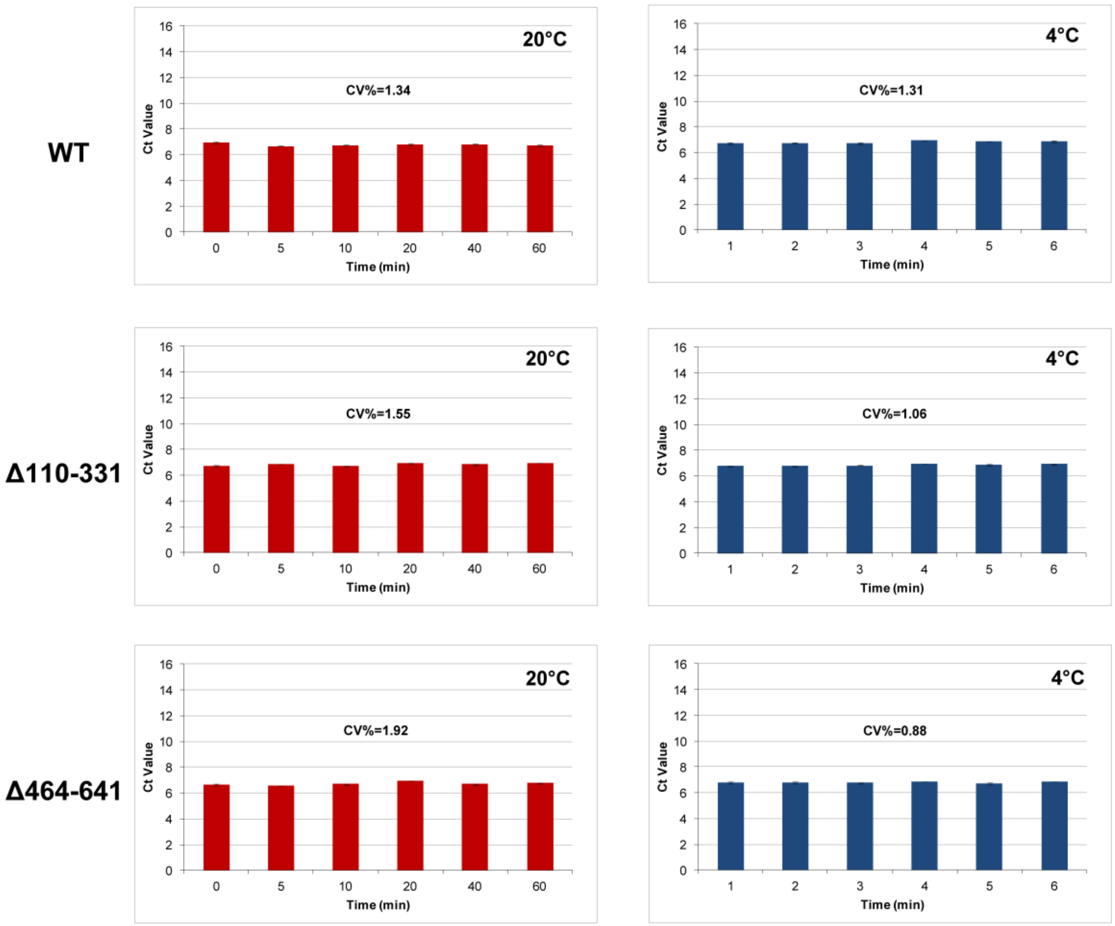

Figure S1. Stability of 16s rRNA of WP3 in the RNA decay assays. The total RNA was reverse transcribed and the Ct values of 16S rRNA gene have been detected by real-time qPCR using cDNA. The Coefficient of Variation (CV%) was calculated and shown. As the Ct levels are inversely proportional to the amount of target nucleic acid in the sample, the low CV value (CV%<2) means that the 16S rRNA is stable under the test conditions and it can be used as the reference for RNA decay experiment.

Table S1 Primers used in this study

Name	Sequence (5'-3')	Description
fpsA-B For	GATTGGGCGGCGTTCTGTAT	co-transcription
fpsA-B Rev	ACCGTTTGGGTCGGGTAAGT	co-transcription
fpsB-C For	TATGAAAGACCCTAAAACGCTACA	co-transcription
fpsB-C Rev	CACAATGACAAGGAACCCAAA	co-transcription
fpsC-D For	ACTCTTTGGGTTCCTTGTCATTG	co-transcription
fpsC-D Rev	AAACATCGGCGGGGTCTATT	co-transcription
fpsD-E For	TTAGTGCAATCCACGCAATCT	co-transcription
fpsD-E Rev	ACACCTGTACCAGCAATGAAAGT	co-transcription
fpsE-F For	CTGCGGTAGATTTAGCAAGTGTT	co-transcription
fpsE-F Rev	AGATAATCATCGGGCGGACA	co-transcription
fpsF-G For	TAGAAGAAAACGAAAATGATACGC	co-transcription
fpsF-G Rev	ACCATCCAATAGAAACACCACC	co-transcription
fpsG-H For	GGTGGTGTTTCTATTGGATGGT	co-transcription
fpsG-H Rev	TGCGGTATTAGGTCTGCTTTTAT	co-transcription
fpsRD1 For	TTTTGGGGAGTATTGAGGGG	co-transcription
fpsRD2 For	AACTTCATTTGGTTTTGTTACGC	co-transcription
fpsRD3 For	AGGGGATGCACAGCAGATAT	co-transcription
fpsRD4 For	AATACTGGCCGTCCGCTAA	co-transcription
fpsRD5 For	CACGCCATACGTTAATGAGTCTCT	co-transcription
fpsRD Rev	GTGTATCCACGGCACAACTATCT	co-transcription
fpsAP For	GGTTTGGATTGTTTTGTTCGC	EMSA
fpsAP Rev	CGCAAGCGTACAACCTAAAGA	EMSA
fpsRP For	CGTTCAGGTTTGCTTGTCTTG	EMSA
fpsRP Rev	ACTGGCAATGAGTAAGAAGAG	EMSA
fpsRHis For	GGTCGCGGATCCATGACAATTGGTGAAAATATAAAG	Protein expression
fpsRHis Rev	CGCTGAAAGCTTTTAACCCTGTTTTTTCTTTTCTTT	Protein expression
fpsApPE	AAGGTGTCGGTAAATCCTGTAA	Primer extension
fpsRpPE	TGTCATTCCAAGGCTTTTACGCT	Primer extension
SW1-331-For	TGTGTCCGGAAACTCACTTATTCAGAAGATTC	UTR deletion
SW1-110-Rev	ACAATCCGGAGATCAAACATGCAAGAAAATAG	UTR deletion
SW1-641-For	CTGCTCCGGATCGACTTACCGAACTAGGCCAC	UTR deletion
SW1-464-Rev	TAACTCCGGATGAGTAAGAAGAGATCTTATCT	UTR deletion
fpsART For	GGCCACAACGCTACAATGG	qPCR
fpsART Rev	TGTTGCGCCTTTTCCTTAGCT	qPCR
fpsRRT For	AACCTAGAAACACTTGTCGCTATATCC	qPCR
fpsRRT Rev	TGCTCTCCAAAAACAATTTCATCT	qPCR
fpsA5URTFor	CGGTTGCCAAAACCAATTG	qPCR
fpsA5URTRev	CGCGGTGTTTGCGTAAAAC	qPCR
fpsAR5URTFor	CTTGCCGTTCAGGTTTGCTT	qPCR
fpsAR5URTRev	GCAAGTGACTACAGAGTCCGATTC	qPCR
16SRTFor	CGCGAGGTGGAGCTAATCTC	qPCR
16SRTRev	TCGAGTTGCAGACTCCAATC	qPCR
